# Triptans in prevention of menstrual migraine: a systematic review with meta-analysis

**DOI:** 10.1186/1129-2377-14-7

**Published:** 2013-01-30

**Authors:** Yong Hu, Xiaofei Guan, Lin Fan, Lingjing Jin

**Affiliations:** 1 Department of neurology, Shanghai Tongji Hospital, Tongji University School of Medicine, Xin-Cun Road 389, Shanghai, 200065, China

**Keywords:** Meta-analysis, Systematic review, Frovatriptan, Naratriptan, Zolmitriptan, Menstruation, Migraine, Prophylaxis

## Abstract

Randomized clinical trials (RCT) assessing the efficacy and tolerability of triptans compared with placebo as short-term prophylaxis of menstrual migraine (MM) were systematically reviewed in this study. Triptans, which interfere with the pathogenesis of migraine and are effective in relieving associated neurovegetative symptoms, have been extensively proposed for prevention of menstrual migraine attacks. We searched Cochrane CENTRAL, MEDLINE and EMBASE for randomized, double-blind, placebo-controlled trials on triptans for MM until 1 Oct, 2012. A total of six RCTs were identified. Two authors independently assessed trial’s quality and extracted data. Numbers of participants free from MM per perimenstrual period (PMP), requiring rescue medication, suffering from headache-associated symptoms and experiencing adverse events in treatment and control groups were used to calculate relative risk (RR) and number needed to treat (NNT) with their corresponding 95% confidence interval (CI). A total of 633 participants received frovatriptan 2.5 mg QD, 584 received frovatriptan 2.5 mg BID, 392 received naratriptan 1 mg BID, 70 received naratriptan 2.5 mg BID, 80 received zolmitriptan 2.5 mg BID, 83 received zolmitriptan 2.5 mg TID and 1104 received placebo. Overall, triptans is an effective, short-term, prophylactic treatment of choice for MM. Considering MM frequency, severity and adverse events, frovatriptan 2.5 mg BID and zolmitriptan 2.5 mg TID tend to be the preferred regimens.

## Review

### Introduction

Migraine, a primary headache disorder, is more common in women (14.5-18%) than in men (4.5-6%) [1,2], and it is often associated with sensory symptoms, nausea or vomiting, and disability [3,4]. Female migraineurs frequently experience headache in association with their menstrual cycles [5,6], which is also known as menstrual migraine (MM). MM includes menstrually-related migraine, defined as migraine with menstruation as well as at other times of the cycle, and pure MM, in which migraine occurred only in association with menstruation on or between day −2 to day +3 [7,8]. MM has been reported to be longer, more disabling, less responsive to acute therapy, and more prone to recurrence than non-menstrual migraine attacks. Effective preventive strategies are key for the management of MM [9-12]. Rofecoxib [13], estradiol [14,15], topiramate [16,17], magnesium [18], nimesulide [19] and naproxen sodium [20] have been used in the treatment of migraine.

Triptans, such as frovatriptan, naratriptan and zolmitriptan, are a class of highly selective serotonin receptor agonists, which can interfere with the pathogenesis of migraine and are effective in relieving the associated neurovegetative symptoms [21-23]. They have been recommended as first-line drugs for the treatment of moderate to severe migraine, including MM [24,25]. In recent years, triptans have been extensively proposed for the prevention of menstrual migraine attacks.

To date, no systematic review has been done to investigate the efficacy and tolerability of triptans in the prevention of MM. This review was undertaken to evaluate the efficacy and tolerability of triptans at different doses in randomized controlled trials (RCTs). Our findings may offer an updated reasonable guide for the treatment of MM in clinical practice.

### Methods

#### Studies

We searched the Cochrane Central Register of Controlled Trials, Medline and Embase using the terms “migraine”, “migraine disorders”, “prevention”, “prophylaxis”, ” menstrual”, “menstrually” and “menses”. References of identified studies were further evaluated. Clinical trials before 1 October 2012 were collected for analysis. There was no restriction on language, but we focused on studies that had been conducted in humans and RCTs. Two authors independently searched and selected studies, and then extracted data from each study for further analysis. Disagreements were resolved by discussion with another author. A placebo comparator is essential to confirm the effectiveness of triptans, and the comparisons were done between triptan and placebo. MM was diagnosed according to the criteria developed by the International Headache Society (IHS) or other definitions that conformed in general to IHS diagnostic criteria [8,26]. There was no restriction on MM frequency, duration or type, dose or route of administration, provided the medication could be self-administered. Although there are a few studies suggesting triptans are effective in the treatment of MM [27-30], data of these studies were not included for analysis to minimize potential confounder.

#### Statistical analysis

Methodological quality was assessed using the Oxford Quality Scale [31]. Studies were analyzed using a single dose of a triptan in reducing the incidence of MM, MM severity, need for rescue medicine and adverse events. The effect of the association was expressed as relative risk (or ’risk ratio’, RR) with its corresponding 95% confidence interval (CI). Pooled RR was estimated using the fixed effects. Heterogeneity between studies was tested using the Q statistic. Such heterogeneity was considered statistically significant if a value of P<0.1 was present [32]. Number needed to treat (NNT) with 95% CI was used as absolute measures of benefit, termed number needed to treat to benefit (NNTB), or harm, termed number needed to treat to harm (NNTH). The data included in this meta-analysis might differ slightly from those in previous reports because we treated data in a consistent manner across all trials. STATA version 12.0 (STATA, College Station, TX, USA) metan package (version 1.86) was used for meta-analyses.

### Results

Studies which were included in this study for analysis are shown in Table 1.

**Table 1:** Clinical trials included in this meta-analysis

	**Country**	Patients (n) *	**Triptan**	**Dose**	**Migraine subtype**	**Treatment duration**	**Day of treatment onset**	**Consecutive PMP**
**Silberstein (2004)**[33]	USA	506	frovatriptan	2.5 mg QD or BID	MAM**	6	−2	1
**Brandes (2009)**[7]	International	410	frovatriptan	2.5 mg QD or BID	MM	6	−2	3
**Newman (2001)**[34]	USA	206	naratriptan	1 mg or 2.5mg BID	MAM**	5	−2	4
**Mannix (s1) (2007)**[35]	USA	287	naratriptan	1 mg BID	MRM**	6	−3	4
**Mannix (s2) (2007)**[35]	International	346	naratriptan	1 mg BID	MRM**	6	−3	4
**Tuchman (2008)**[36]	USA	244	zolmitriptan	2.5 mg BID or TID	MM**	7	−2	3

* intention-to-treat population.

** used Headache Society (IHS) 1988 criteria.

#### Studies

Seven trials that met our inclusion criteria were identified, among which one [37] was excluded because its data were included in one of the remaining 6 trials. All the six trials (Table 1) were double-blind. Among them, Silberstein’s trial (2004) was cross-over in design. All of these studies focused on a single dose of a triptan in the prevention of MM and were multicentred. The mean age of participants ranged from 36 to 38 years, and all were women.

All trials compared a triptan with placebo. No trials directly compared one drug with another. Frovatriptan 2.5 mg QD and 2.5 mg BID were tested in two trials, naratriptan 1 mg BID in three trials, and zolmitriptan in one trial. In Newman’s trial (2001), two doses of naratriptan (1 mg, 2.5 mg) were administered. A total of 633 participants received frovatriptan 2.5 mg QD, 584 received frovatriptan 2.5 mg BID, 392 received naratriptan 1 mg BID, 70 received naratriptan 2.5 mg BID, 80 received zolmitriptan 2.5 mg BID, 83 received zolmitriptan 2.5 mg TID and 1104 received placebo.

Most trials evaluated the proportion of patients without MM during the treated PMPs as the primary outcome. Three of six trials evaluated the proportion of patients using rescue medication and two evaluated the severity of MM. Two of six trials evaluated the MM duration and MM associated symptoms in distinct ways.

**Figure 1:**
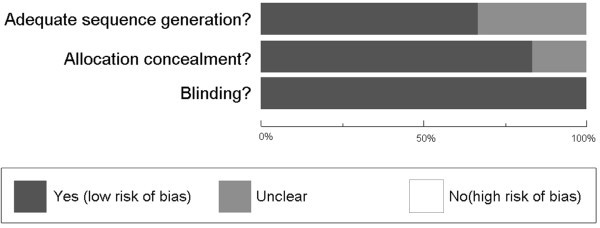
Methodological quality assessment: methodological quality presented as percentage across all studies.

Methodological quality, assessed using the Oxford Quality Scale, was good in all studies. Except Tuchman’s trial (2008) which scored 3/5 and Newman’s trial (2001) which scored 4/5, all the other trials were scored 5/5. Points were lost due to failure to adequately report the method of randomization and blinding. All studies reported withdrawals or dropouts. Sequence generation, allocation concealment and blinding were assessed using the “risk of bias” tool (Figure 1). No studies were at high risk of bias.

#### Primary outcomes

For the primary outcomes of interest, we focused on the proportion of patient free from MM during the treated PMPs. Except Silberstein (2004), all the other trials crossed more than one PMP, and thus being free from MM per PMP was selected as our primary outcome. The characteristics of MM including MM severity were used as secondary outcomes.

#### Frovatriptan

**Figure 2:**
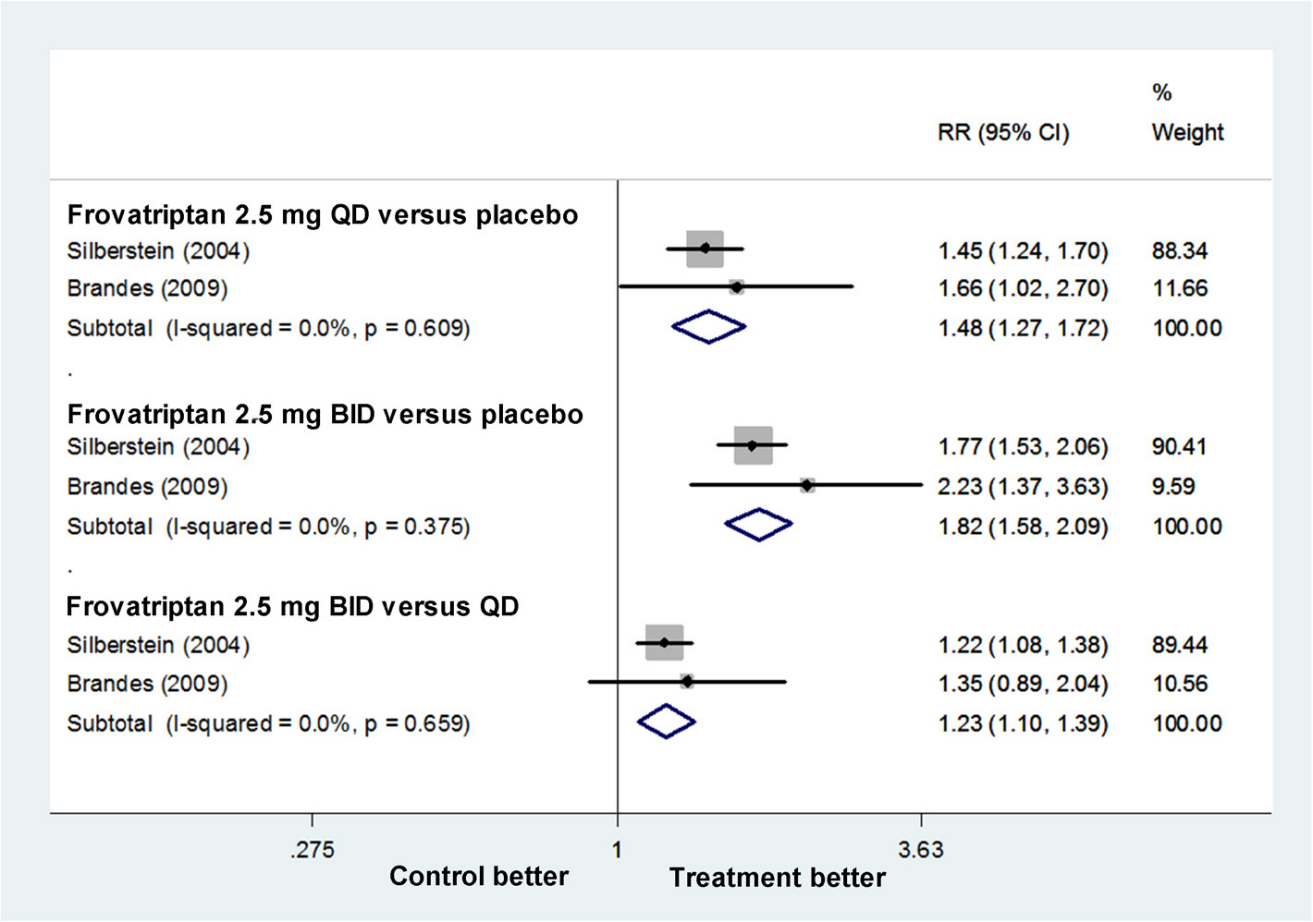
Forest plot: free from MAM per PMP in trials on frovatriptan.

Silberstein (2004) and Brandes (2009) tested frovatriptan 2.5 mg QD and BID. The relative benefit of frovatriptan 2.5 mg QD compared with placebo was 1.48 (1.27 to 1.72), giving an NNTB of 7.22 (5.25 to 11.54); that of frovatriptan 2.5 mg BID compared with placebo was 1.82 (1.58 to 2.09), giving an NNTB of 3.90 (3.23 to 4.93). Patients with frovatriptan 2.5 mg BID had a 23% increase in free from MM per PMP 1.23 (1.10 to 1.39), giving an NNTB of 8.50 (5.77 to 16.19), compared with frovatriptan 2.5 mg QD (Figure 2).

#### Naratriptan

**Figure 3:**
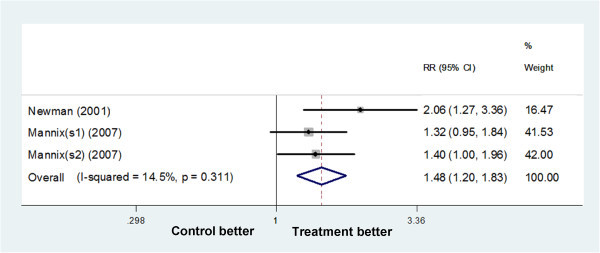
Forest plot: free from MAM per PMP in trials on naratriptan 1 mg BID.

Three trials (Mannix (s1) (2007), Mannix (s2) (2007), Newman (2001)) tested naratriptan 1 mg BID. The relative benefit of naratriptan compared with placebo was 1.48 (1.20 to 1.83), giving an NNTB of 7.98 (5.24 to 16.71) (Figure 3). Only Newman (2001) using naratriptan 2.5 mg BID reported that naratriptan treated patients had fewer overall MMs and fewer MM days compared with patients in the placebo group, however no significant differences were found. The NNT for naratriptan 2.5 mg BID was not calculated.

#### Zolmitriptan

Tuchman (2008), using zolmitriptan 2.5 mg BID and TID reported that both zolmitriptan regimens demonstrated superior efficacy vs placebo, as measured by ≥50% reduction in the frequency of MM and the mean number of breakthrough MM per menstrual cycle. There were insufficient data for meta-analysis. The NNTBs for free from MM per menstrual cycle zolmitriptan 2.5 mg BID versus placebo, 2.5 mg TID versus placebo and 2.5 mg TID versus BID were 4.98 (3.26 to 10.57), 2.52 (1.95 to 3.58) and 5.11 (2.95 to 18.93) respectively.

All studies had scores of methodological quality of ≥ 3/5, and no sensitivity analysis for primary outcomes was carried out for this criterion.

#### Secondary outcomes

For the secondary outcomes of interest, we focused on MM severity, need for rescue medication, adverse events.

#### Frovatriptan

**Figure 4:**
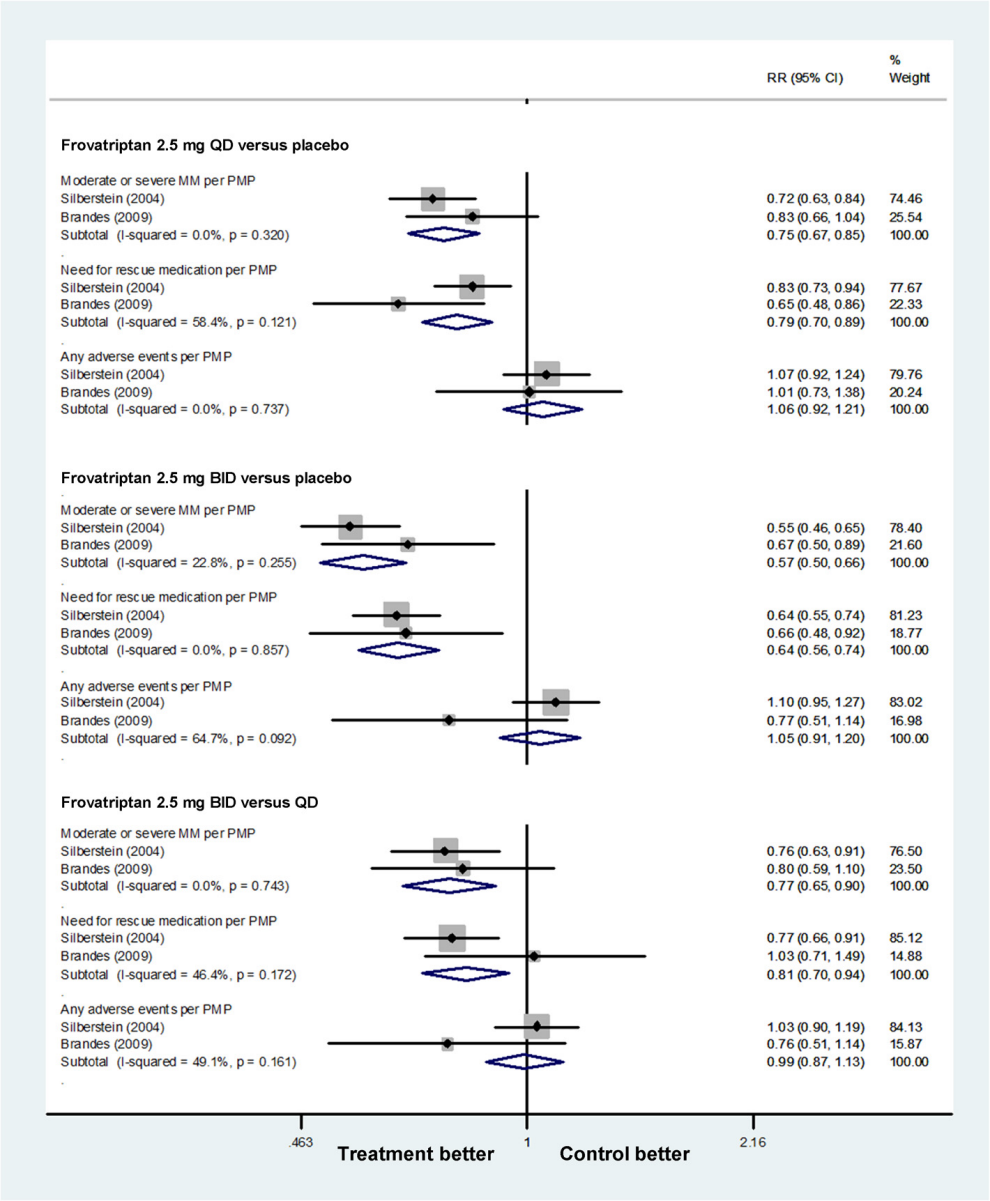
Forest plot: secondary outcomes of frovatriptan.

Patients with frovatriptan, both 2.5 mg QD and BID, had a reduction in MM severity and need for rescue medication, and BID was superior to QD. Frovatriptan 2.5 mg QD had a reduction in moderate to severe MM per PMP (0.75 [0.67 to 0.85]) giving an NNTB of 7.70 (5.43 to 13.19), and in need for rescue medication per PMP (0.79 [0.70 to 0.89]) giving an NNTB of 9.28 (6.17 to 18.72) when compared with placebo. Analogously, frovatriptan 2.5 mg BID had a reduction in moderate to severe MM per PMP (0.57 [0.50 to 0.66]) giving an NNTB of 4.43 (3.58 to 5.81), and in need for rescue medication per PMP (0.64 [0.56 to 0.74]) giving an NNTB of 5.57 (4.28 to 7.99) when compared with placebo. Frovatriptan 2.5 mg BID vs QD had a reduction in moderate to severe MM per PMP (0.77 [0.65 to 0.90]) giving an NNTB of 10.45 (6.72 to 23.44), and in need for rescue medication per PMP (0.81 [0.70 to 0.94]) giving an NNTB of 13.93 (7.94 to 56.73) (Figure 4).

The adverse events in frovatriptan 2.5 mg QD vs placebo, frovatriptan 2.5 mg BID vs placebo and frovatriptan 2.5 mg QD vs BID were comparable. Most reported adverse events were mild to moderate. The incidence of severe adverse events was low and appeared to be unrelated to the treatments [7,33].

#### Naratriptan

**Figure 5:**
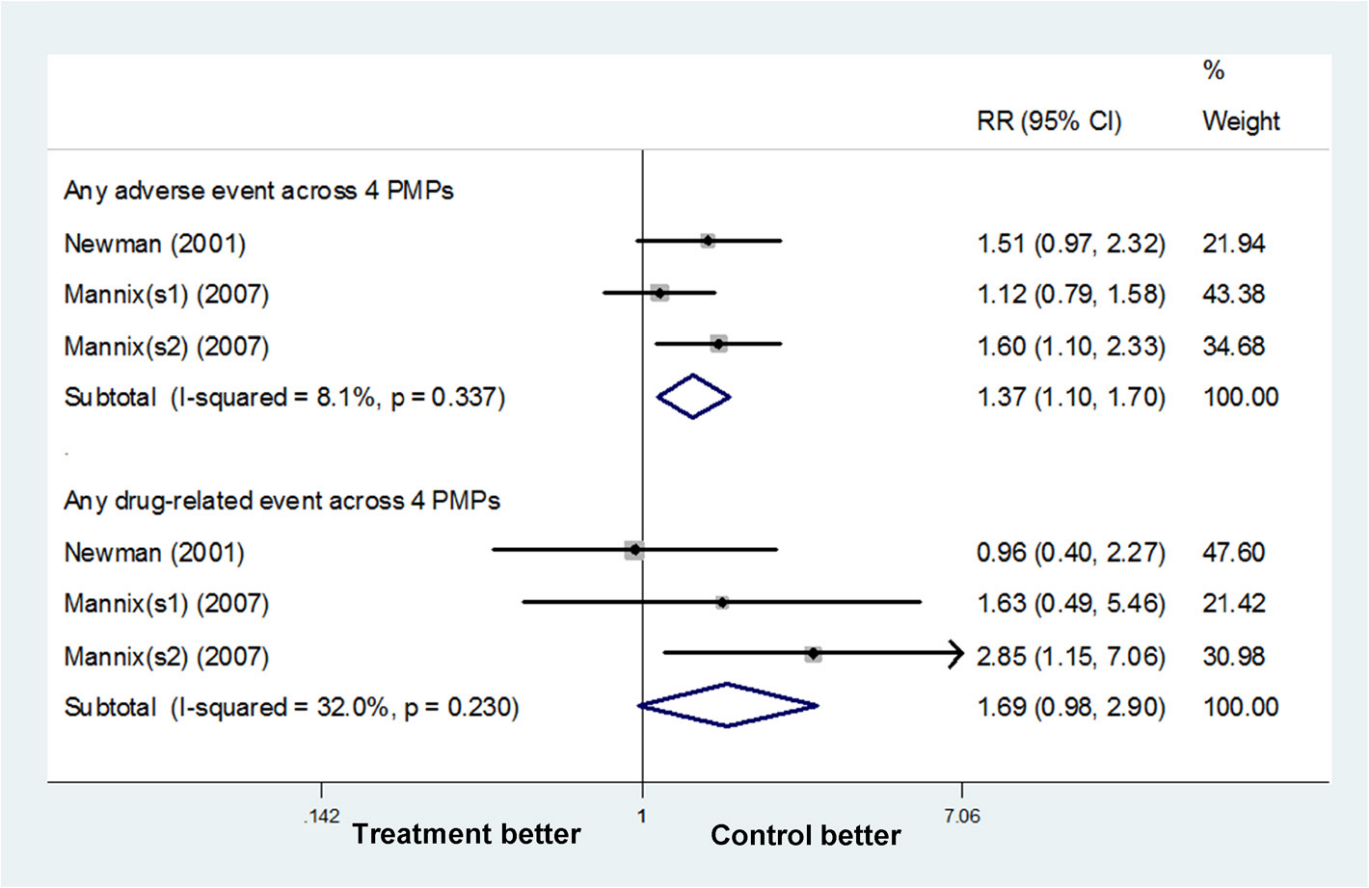
Forest plot: secondary outcomes of naratriptan 1 mg BID vs placebo.

Because the changes in MM severity and need for rescue medication were not mentioned in trails on naratriptan, we focus on adverse events and drug-related events as the secondary outcomes of trials on naratriptan. After treatment with naratriptan 1 mg BID, there was an increase in adverse events (1.37 [1.10 to 1.70]) giving an NNTH of 10.88 (6.46 to 34.38), but drug-related events (1.69 [0.98 to 2.90]) were comparable to the placebo (Figure 5). Newman (2001) using naratriptan 2.5 mg BID reported that the overall adverse events and drug-related events were similar to those in placebo group. In all studies, serious drug-related adverse events were not reported [34,35].

#### Zolmitriptan

Tuchman (2008) reported both zolmitriptan 2.5 mg BID (0.82 [0.71 to 0.94], giving an NNTB of 7.31 [4.32 to 23.81]) and zolmitriptan 2.5 mg TID (0.83 [0.71 to 0.97], giving an NNTB of 7.81 [4.31 to 41.64]) demonstrated a reduction in the need for rescue medication when compared with placebo.

**Figure 6:**
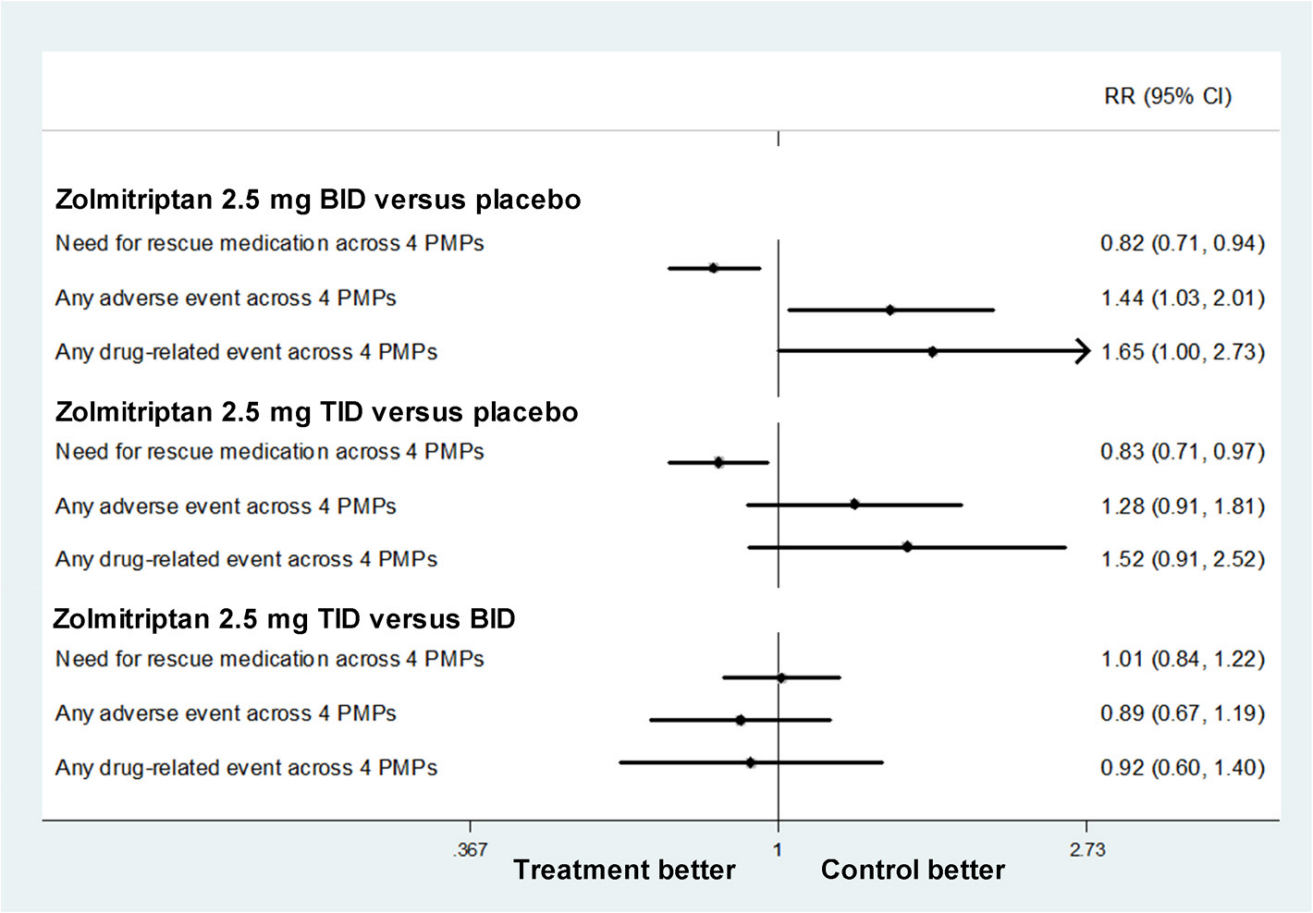
Forest plot: secondary outcomes of Zolmitriptan.

Zolmitriptan 2.5 mg BID had an increase in any adverse event across 4 PMPs (1.44 [1.03 to 2.01]), giving an NNTH of 7.81 (4.31 to 41.64) when compared with placebo (Figure 6). Five serious adverse events were reported during the preventative therapy: two in the zolmitriptan 2.5 mg TID group (pyelonephritis and endometrial disorder), two in the zolmitriptan 2.5 mg BID (uterine neoplasm and anxiety) and one in the placebo group [36]. When drug-related adverse events were valued, no significant difference was found between treatment group and control group.

Data of specific adverse events was too few for analysis and was not consistently reported in these studies, so we just list the most commonly adverse events for all the three triptans in Table 2.

**Table 2:** Incidence of most commonly reported adverse events

	**Treatment**	**Placebo**
**Frovatriptan 2.5 mg QD**	**n=501**	**n=505**
Nausea	4.80%	3.40%
Headache	4.60%	6.30%
Dizziness	3.60%	2.60%
Nasopharyngitis	3.20%	2.40%
Dysmenorrhea	2.20%	3.00%
**Frovatriptan 2.5 mg BID**	**n=501**	**n=505**
Nausea	6.80%	3.40%
Dizziness	4.80%	2.60%
Headache	4.20%	6.30%
Nasopharyngitis	3.40%	2.40%
Dysmenorrhea	1.80%	3.00%
**Naratriptan 1 mg BID**	**n=71**	**n=68**
Dyspeptic symptoms	4.23%	0.00%
Malaise and fatigue	1.41%	2.94%
Dizziness	1.41%	1.47%
Hyposalivation	1.41%	0.00%
Parasthesia	1.41%	0.00%
**Naratriptan 2.5 mg BID**	**n=71**	**n=68**
Dizziness	4.23%	1.47%
Chest symptoms	2.82%	2.94%
Malaise and fatigue	2.82%	2.94%
Hyposalivation	2.82%	0.00%
Parasthesia	2.82%	0.00%
Burning/stinging sensations	2.82%	0.00%
**Zolmitriptan 2.5 mg BID**	**n=80**	**n=82**
Asthenia	8.75%	9.76%
Dizziness	6.25%	4.88%
Somnolence	6.25%	3.66%
Nausea	6.25%	1.22%
Tightness	5.00%	2.44%
Headache	3.75%	2.44%
Dry mouth	1.25%	1.22%
**Zolmitriptan 2.5 mg TID**	**n=84**	**n=82**
Asthenia	10.71%	9.76%
Headache	8.33%	2.44%
Dizziness	7.14%	4.88%
Somnolence	7.14%	3.66%
Tightness	7.14%	2.44%
Nausea	7.14%	1.22%
Dry mouth	5.95%	1.22%

### Discussion

#### Summary of main results

In this systematic review, six trials were included, and we compared the frovatriptan, naratriptan and zolmitriptan at different doses with placebo in preventing MM. We employed explicit and systematic methods to identify, select and critically appraise studies, and to extract data followed by a meta-analysis. Frovatriptan was given to 633 participants at 2.5 mg QD and to 584 participants at 2.5 mg BID and 646 participants were treated with placebo. Naratriptan was given to 462 participants at 1 mg or 2.5 mg BID who were compared with 377 placebo treated participants. In addition, 80 participants received zolmitriptan 2.5 mg BID, 83 received zolmitriptan 2.5 mg TID and 81 received placebo. In all trails, triptans were for 5–6 days at 2–3 days before MM onset. Overall, triptans were more effective than placebo in the prevention of MM.

All of the three triptans, including frovatriptan naratriptan and zolmitriptan, were more effective than placebo in reducing the MM frequency per PMP. The incidence of moderate to severe MM per PMP and the need for rescue medication occurred less often with frovatriptan than with placebo. Patients with zolmitriptan had less need for rescue medicine than placebo treated patients. One of the putative benefits of preventative therapy for MM with triptans is to reduce the severity of MM attacks. As the NNTBs for frovatriptan 2.5 mg BID, naratriptan 1 mg BID, zolmitriptan 2.5 mg TID in reducing the incidence of MM per PMP were 3.90 (3.23 to 4.93), 7.98 (5.24 to 16.71) and 2.52 (1.95 to 3.58), respectively, zolmitriptan and frovatriptan tended to be more effective than naratriptan.

Except frovatriptan, adverse events were more common in triptans treated patients. However, when drug-related events were taken into account, there were no significant difference between triptans group and placebo group. Adverse events were generally mild to moderate and rarely led to withdrawal. Studies on triptan at one or two doses were underpowered for investigating the specific adverse events, and studies with longer duration are required to determine the adverse event profiles of triptans in the treatment of MM. However, the safety and tolerability of triptans in preventing migraine had been well established. Considering MM frequency and adverse events, frovatriptan 2.5 mg BID and zolmitriptan 2.5 mg TID are preferred for the prevention of MM.

One interesting finding was that the efficacy of triptans in prevention migraine was dependent on their concentrations. There were insufficient data to establish a clear dose–response curve. However, frovatriptan 2.5 mg BID tended to be better than frovatriptan 2.5 mg QD in reducing headache during PMP, and zolmitriptan 2.5 mg TID better than zolmitriptan 2.5 mg BID. These suggest the efficacy of triptans is concentration and time-dependent. Does it mean that higher dose or frequency will bring better effect? In trials on naratriptan which has a half-time of 6 h [35], naratriptan 1 mg BID was statistically superior to naratriptan 2.5 mg BID. This implies that the efficacy of triptans in prevention of migraine depends on their concentrations. Considering that frovatriptan has a long half-life (26 h [33]) and zolmitriptan posses a short half-life (3 h [38]), the frequency and dose should be adjusted when a triptan is used in the prevention of MM.

#### Overall completeness and applicability of evidence

Interpretation of these results is limited by the small number of studies (n=6) which only included three drugs at two doses. There were inadequate numbers of participants and events to draw firm conclusions about possible differences among different drugs or doses.

Because the enlistment of trials was through clinics, this is likely to underestimate the therapeutic effect. Clinics may select participants whose MM are more severe, resistant to treatment than in the general population. On the other hand, these participants may be more motivated than the population as a whole.

In the available trials, triptans were used at safe doses while still experiencing clinically useful levels of efficacy. This may mean limitation on effective treatment; maybe combined therapy with adjunctive drugs, such as estradiol or topiramate, might increase the effectiveness.

In this review, data related to migraine attacks between menses were not identified. This overlooks the possibility, even very small, that preventative therapy may delay the attacks after treatment. Further studies are needed to monitor migraine attacks in menses and to establish the differences among different triptans and different doses. Long-term studies are also required to determine the safety and tolerability. Further studies comparing triptans with alternative drugs, such as estradiol or topiramate, are allowable to determine the relative benefits and harms.

The cost of triptans for MM prevention should also be taken into consideration. Each triptan is much more expensive than other prophylactic medications for migraine (such as beta blockers, tricyclic antidepressants, sodium valproate, and methysergide) [39]. At the same time, menstrual cycles have natural variability, and even in women whose menstrual cycles are regular, very few invariably attacks have associated with their periods. These infectors inevitably expose women to the use of medication during cycles in which they would not have a headache. Thus, only MM women who do not achieve adequate relief from acute therapies may become candidates for short-term preventative therapy with a triptan, particularly if they experience regular menstrual migraine attacks causing significant disability. In this way, MM attack frequency is decreased, and these patients have the opportunity to regain the days lost each month through disability.

#### Quality of the evidence

All studies were good in methodological quality and scored above the minimum required to minimize bias. IHS criteria or other definitions that generally conformed to IHS diagnostic criteria were used for the diagnosis of MM in all trials, and well-defined outcomes were reported for the efficacy and tolerability.

## Conclusion

Current RCTs suggest that triptans treatment is an effective, short-term, prophylactic strategy for the management of MM. Considering MM frequency, severity and adverse events, frovatriptan 2.5 mg BID and zolmitriptan 2.5 mg TID are preferred for the treatment of MM.

## Competing interest

The authors declare that there is no conflict of interest.

## Authors’ contributions

LJ and YH designed this study. YH and XG carried out the searches, identified studies for inclusion and extracted relevant data. YH, XG and LF were involved in analysis. LJ acted as arbitrator. All authors saw and approved the final version.

## Open access

This article is distributed under the terms of the Creative Commons Attribution License which permits any use, distribution, and reproduction in any medium, provided the original authors and the source are credited.
